# Immunometabolic regulation in gouty arthritis: current evidence, mechanistic insights, and remaining challenges

**DOI:** 10.3389/fimmu.2026.1795337

**Published:** 2026-07-06

**Authors:** Xingzheng Liu, Xingrui Yan, Tao Wang, TingTing Luo, Shengqin Yang, Yixin Fang, Xiumin Chen, Runyue Huang, Maojie Wang, Xiaodong Wu

**Affiliations:** 1 State Key Laboratory of Traditional Chinese Medicine Syndrome, The Second Affiliated Hospital of Guangzhou University of Chinese Medicine (Guangdong Provincial Hospital of Chinese Medicine), The Second Clinical Medical College of Guangzhou University of Chinese Medicine, Guangzhou, China; 2 Guangdong Provincial Key Laboratory of Clinical Research on Traditional Chinese Medicine Syndrome, Guangzhou, China; 3 Guangdong-Hong Kong-Macau Joint Lab on Chinese Medicine and Immune Disease Research, Guangzhou University of Chinese Medicine, Guangzhou, China; 4 Guangdong Provincial Key Laboratory of Chinese Medicine for Prevention and Treatment of Refractory Chronic Diseases, Guangzhou, China

**Keywords:** gouty arthritis, immunometabolism, macrophages, neutrophils, NLRP3 inflammasome

## Abstract

Gouty arthritis is driven by monosodium urate (MSU) crystal deposition and acute activation of the NLRP3 inflammasome–IL-1β axis. However, crystal burden alone does not fully explain asymptomatic crystal deposition, recurrent flares, or the self-limiting nature of acute inflammation. Immunometabolism provides a useful perspective for understanding these differences. In macrophages, glycolysis supports pro-IL-1β expression and inflammatory mediator production, while tricarboxylic acid cycle remodeling, mitochondrial stress, and reactive oxygen species may contribute to inflammasome activation. In neutrophils, glycolysis sustains rapid effector functions, including chemotaxis, degranulation, ROS production, and neutrophil extracellular trap formation. During later stages, efferocytosis, lipid mediator switching, and mitochondrial adaptation may promote inflammation resolution. Systemic metabolic abnormalities may further influence recurrence susceptibility by altering innate immune responsiveness to MSU crystals. This review summarizes current evidence linking immunometabolic pathways to gouty inflammation and discusses remaining questions regarding cell specificity, temporal sequence, causal relevance, and therapeutic translation.

## 1 Introduction

Gout is a systemic urate crystal deposition disease that develops in the setting of sustained hyperuricemia, whereas gouty arthritis refers specifically to the inflammatory joint manifestations induced by monosodium urate (MSU) crystals (1). Acute gouty arthritis typically presents with sudden joint redness, swelling, warmth, and pain (2). Recurrent flares may lead to chronic tophaceous gout, characterized by bone erosion, joint deformity, and functional impairment. Beyond articular involvement, gout is closely associated with obesity, metabolic syndrome, chronic kidney disease, and cardiovascular comorbidities, suggesting that it represents a systemic disorder involving coordinated interactions among urate metabolism, crystal deposition, metabolic dysregulation, and innate immune activation (3, 4). In this review, gouty arthritis refers specifically to joint inflammation, whereas gout denotes systemic disease features.

The core process of MSU crystal-induced innate immune inflammation is increasingly well characterized. After phagocytosis by monocytes, macrophages, and other immune cells, MSU crystals trigger lysosomal damage, potassium efflux, mitochondrial stress, and activation of the NLRP3 inflammasome, culminating in caspase-1 activation and maturation of IL-1β (2, 5). IL-1β subsequently acts on synovial fibroblasts, vascular endothelial cells, and other immune cells, inducing chemokines, adhesion molecules, and inflammatory mediators, thereby promoting neutrophil recruitment and local amplification of inflammation (6, 7). This MSU crystal–NLRP3 inflammasome–IL-1β axis forms the mechanistic basis of acute gout flares and underpins current anti-inflammatory therapeutic strategies (8).

However, crystal deposition does not correspond directly with clinical flares (9). Some individuals with MSU crystal deposition remain asymptomatic for extended periods, whereas others experience recurrent acute flares under similar hyperuricemic or crystal-laden conditions. Acute gout inflammation is often self-limiting, as local inflammation typically resolves within several days even without targeted therapy (10). These observations suggest that while MSU crystals are necessary for gout inflammation, the initiation, amplification, resolution, and recurrence of inflammation are also modulated by host response states and the local tissue microenvironment (11).

Immunometabolism offers an additional perspective on these regulatory processes (12). Immune cell metabolism not only provides energy and biosynthetic precursors for cellular activation but also modulates inflammatory gene expression, inflammasome activation, redox balance, lipid mediator production, and apoptotic cell clearance, thereby influencing the intensity and duration of inflammation. Compared with previous reviews focusing on either urate metabolism or inflammasome signaling, this review integrates macrophage- and neutrophil-centered immunometabolic programs across the entire trajectory of gouty inflammation, from crystal sensing to resolution and recurrence susceptibility.

## 2 MSU crystal-induced innate inflammatory axis

The innate immune response triggered by MSU crystals represents the most well-characterized mechanistic basis for acute gout flares and provides the primary framework for understanding immunometabolic changes in gout. Immunometabolic alterations primarily influence crystal recognition, inflammasome activation, IL-1β production, and neutrophil effector responses, thereby modulating the intensity and duration of inflammation (13). Although clinical manifestations of gout are affected by uric acid levels, crystal burden, local tissue environment, and host factors (14), the NLRP3 inflammasome and IL-1β signaling remain central for linking MSU crystal stimulation to the amplification of acute joint inflammation (6).

### 2.1 NLRP3 inflammasome and IL-1β release

After MSU crystal deposition in the joint cavity or synovial tissue, crystals are phagocytosed by local macrophages, monocytes, and other innate immune cells. Within the cell, MSU crystals do not trigger inflammation via a single receptor; rather, multiple intracellular stress signals converge to activate the NLRP3 inflammasome (15). Among these, lysosomal damage is an early and critical event. Due to the strong physical properties of the crystals, phagocytosed MSU crystals can destabilize lysosomal membranes, releasing proteases and other contents into the cytoplasm, thereby providing danger signals that promote inflammasome assembly.

In addition to lysosomal damage, potassium efflux, disturbances in intracellular ionic homeostasis, and mitochondrial stress contribute to NLRP3 activation (5). MSU crystal stimulation can induce mitochondrial dysfunction, increased mitochondrial reactive oxygen species (mtROS), and release of mitochondrial DNA. These changes do not imply that enhanced mitochondrial metabolism alone drives gout flares; rather, they reflect the cumulative effect of organelle stress, oxidative stress, and inflammatory signaling following crystal uptake. Under conditions of combined lysosomal and mitochondrial stress, NLRP3, ASC, and pro-caspase-1 more readily assemble into the inflammasome complex.

Activation of the NLRP3 inflammasome leads to cleavage of pro-caspase-1 into active caspase-1, which then processes pro-IL-1β into mature IL-1β (16). IL-1β is one of the key mediators of acute gouty inflammation, marking the conversion of intracellular crystal recognition into tissue-level inflammatory responses. Mature IL-1β acts on synovial fibroblasts, endothelial cells, and other immune cells, inducing the expression of inflammatory mediators and chemokines such as IL-6, CXCL8, and CCL2, while promoting upregulation of adhesion molecules on vascular endothelial cells, thereby facilitating recruitment of circulating neutrophils to the joint (17).

### 2.2 Neutrophil recruitment and acute inflammatory amplification

Once neutrophils infiltrate the joint cavity, the inflammatory response is further amplified. Neutrophils participate in local inflammation through phagocytosis of MSU crystals, protease release, ROS generation, and formation of neutrophil extracellular traps (NETs) (17). Consequently, gout inflammation progresses from early crystal recognition and inflammasome activation within a few phagocytic cells to widespread acute joint inflammation characterized by massive immune cell infiltration, cytokine release, and tissue responses. This mechanistic process explains the sudden onset, severe pain, and rapid escalation of local inflammation observed in acute gout flares.

Although this classical inflammatory axis provides a clear mechanistic framework for acute gout, crystal deposition alone does not always trigger clinical inflammation. Some individuals with MSU crystal deposition remain asymptomatic for long periods, while others experience recurrent acute flares. Flares in the same patient often show temporal variability, occurring after long intervals without significant changes in crystal burden. These observations suggest that MSU crystals are necessary for gout inflammation but not the sole determinant (9, 11). Whether crystal stimulation translates into clinical flares likely depends on multiple factors, including the activation state of local immune cells, the synovial microenvironment, systemic metabolic background, and the efficiency of inflammation resolution.

**Figure 1:**
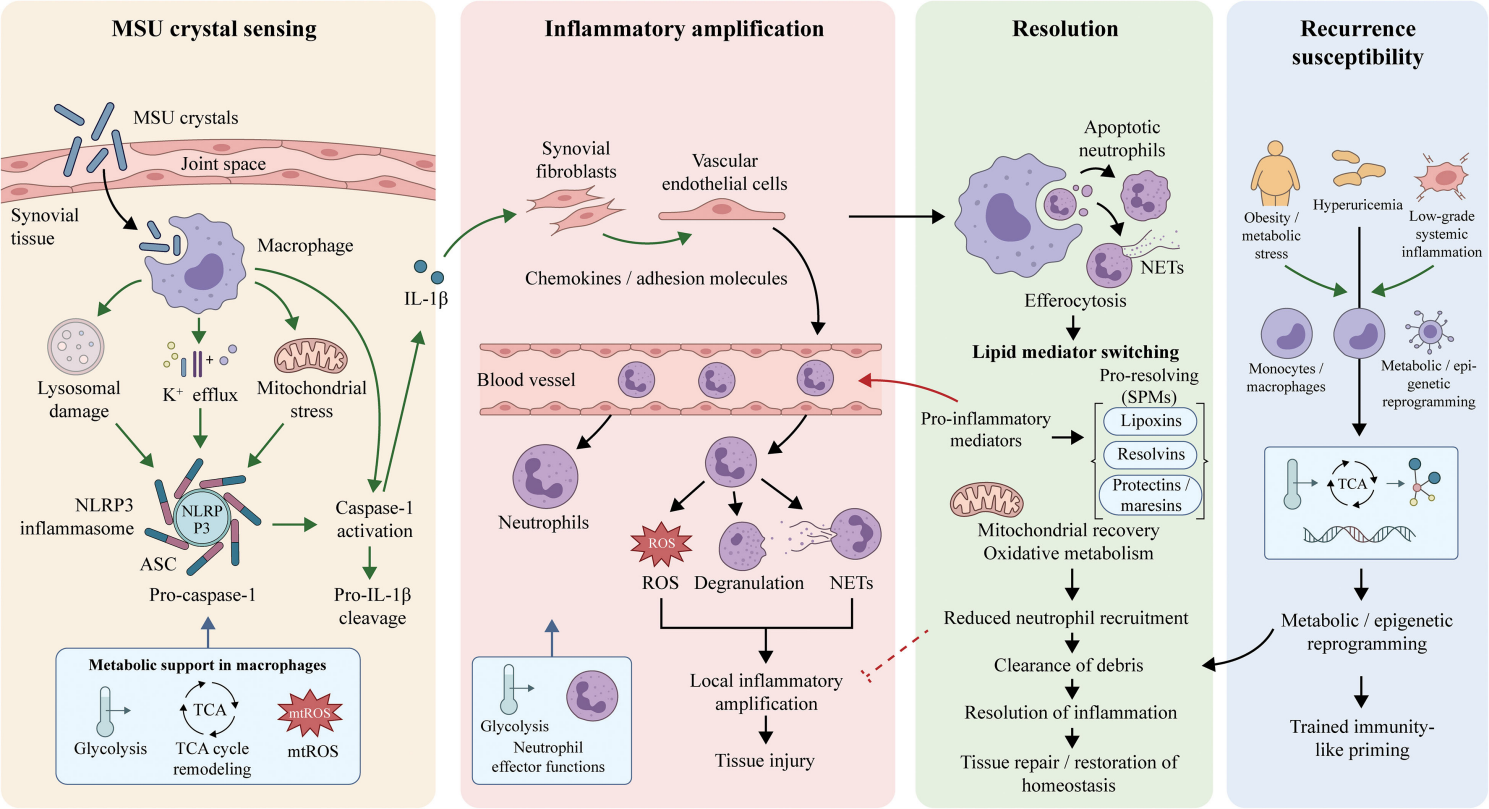
Overview of immunometabolic regulation in gouty arthritis. MSU crystals deposited in the joint are phagocytosed by macrophages and induce lysosomal damage, potassium efflux, mitochondrial stress, NLRP3 inflammasome activation, caspase-1 activation, and IL-1β release. IL-1β promotes chemokine production and adhesion molecule expression by synovial fibroblasts and vascular endothelial cells, thereby facilitating neutrophil recruitment and inflammatory amplification through ROS production, degranulation, and NET formation. During resolution, macrophage efferocytosis, lipid mediator switching toward specialized pro-resolving mediators, mitochondrial recovery, and debris clearance contribute to reduced neutrophil recruitment, resolution of inflammation, and restoration of tissue homeostasis. Systemic metabolic stress, hyperuricemia, and low-grade inflammation may alter monocyte/macrophage responsiveness through metabolic and epigenetic reprogramming, potentially contributing to recurrence susceptibility.

In this context, immunometabolism is best understood as a cell-intrinsic regulatory mechanism modulating the response to MSU crystals, influencing inflammasome activation, inflammatory amplification, and resolution. Specifically, macrophage metabolic states affect pro-IL-1β expression, NLRP3 inflammasome assembly, and cytokine release; neutrophil metabolism is closely associated with chemotaxis, degranulation, ROS production, and NET formation. During later stages of inflammation, metabolic adaptations may also support clearance of apoptotic cells, production of pro-resolving lipid mediators, and tissue repair (12). Together, these processes provide the framework for discussing how immunometabolic pathways regulate macrophage activation, neutrophil effector responses, inflammation resolution, and recurrence susceptibility in gouty arthritis (**Figure 1**).

## 3 Macrophage immunometabolism and gouty inflammation

Macrophages are key cellular mediators of MSU crystal-induced inflammatory responses. They not only phagocytose MSU crystals and initiate NLRP3 inflammasome activation and IL-1β release, but also participate in apoptotic cell clearance and tissue repair during the later stages of inflammation (15). Thus, macrophage function in gout is not fixed; rather, it varies according to local stimuli, inflammatory stage, and metabolic environment (12). Recent studies suggest that MSU crystal stimulation can induce metabolic reprogramming in macrophages, characterized by GLUT1-mediated glucose uptake and enhanced aerobic glycolysis, thereby influencing NLRP3 inflammasome activation and IL-1β release (18). In addition, tricarboxylic acid (TCA) cycle intermediates (19), mitochondrial stress (20), and lipid-related metabolic signals (21) have also been proposed to modulate the magnitude of macrophage inflammatory responses. It should be noted, however, that these metabolic changes should not be simply interpreted as the initiating events of gout flares. In most existing studies, metabolic reprogramming has been observed after MSU crystal, uric acid, or inflammatory stimulation, and NLRP3/IL-1β signaling itself may also reciprocally regulate glycolysis (22). Therefore, these metabolic alterations may contribute to inflammatory amplification, but may also represent accompanying changes during the inflammatory process.

### 3.1 Glycolysis and IL-1β production

Upon inflammatory stimulation, macrophages often shift from a predominantly mitochondrial oxidative metabolic state toward increased reliance on glycolysis (18). Similar changes have also been observed in MSU crystal-associated inflammation. Enhanced glycolysis provides ATP for rapid cellular activation and supplies metabolic intermediates for nucleotide, amino acid, and lipid biosynthesis, thereby supporting cytokine production and functional changes in macrophages.

In acute gouty inflammation, IL-1β production generally involves two steps. First, macrophages upregulate NLRP3 and pro-IL-1β expression through signaling pathways such as NF-κB. Subsequently, MSU crystal-induced lysosomal damage, ionic fluxes, and mitochondrial stress promote inflammasome assembly and caspase-1 activation. Enhanced glycolysis mainly contributes to the first step by increasing inflammatory gene expression and protein synthesis capacity (23). Increased glucose uptake, elevated glycolytic flux, and lactate production may facilitate the transition of macrophages into an inflammatory state and provide metabolic support for IL-1β production (24).

Some glycolytic enzymes also regulate inflammation through non-canonical functions. For example, pyruvate kinase M2 can undergo conformational and localization changes in inflammatory macrophages and interact with transcriptional regulators such as HIF-1α, thereby promoting the expression of inflammatory genes, including IL-1β (25, 26). Glyceraldehyde-3-phosphate dehydrogenase (GAPDH) can also influence cytokine production by regulating the translation of inflammatory mRNAs (27). When glycolytic activity increases, GAPDH is preferentially engaged in metabolic reactions, reducing its translational repression of selected inflammatory mRNAs and thereby favoring inflammatory protein expression (28).

Therefore, enhanced glycolysis is more appropriately viewed as a metabolic program that supports MSU crystal-induced inflammatory output, rather than as a gout-specific trigger that independently determines flare initiation. Its major significance lies in providing metabolic support for pro-IL-1β expression, inflammatory mediator synthesis, and rapid macrophage activation.

### 3.2 TCA cycle remodeling, mitochondrial stress, and NLRP3 activation

Inflammatory activation of macrophages is often accompanied by remodeling of the TCA cycle, leading to dynamic changes in metabolites such as citrate, succinate, fumarate, and itaconate. These metabolites not only participate in energy metabolism but also regulate inflammatory signaling, redox homeostasis, and gene expression (29, 30). Among them, succinate can accumulate in LPS-activated macrophages and promote IL-1β expression by stabilizing HIF-1α (19). Succinate oxidation can also enhance mtROS production through succinate dehydrogenase and the mitochondrial electron transport chain, while mtROS represents an important signal involved in NLRP3 inflammasome activation (31, 32).

**Figure 2:**
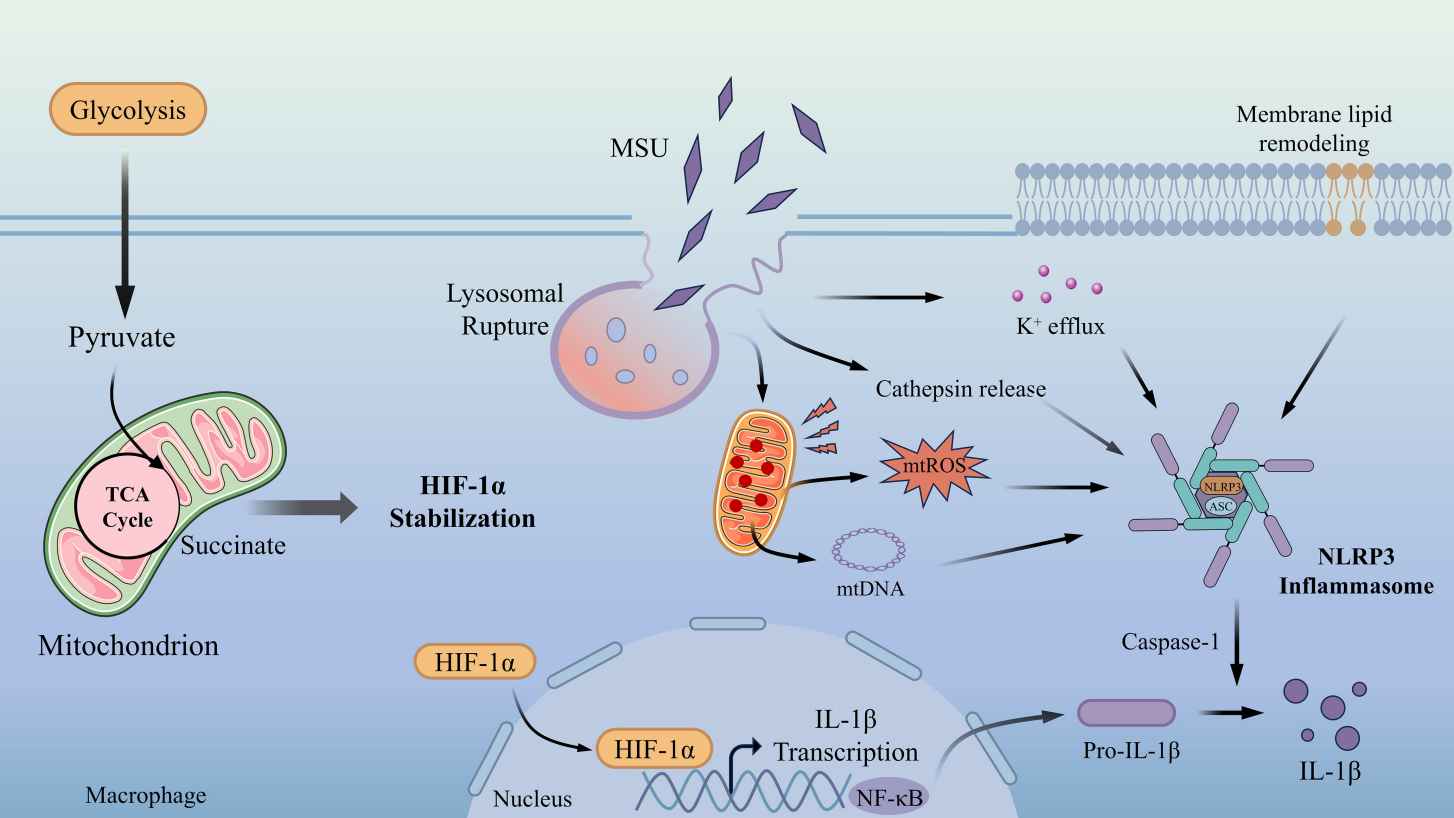
Macrophage immunometabolism in MSU crystal-induced inflammasome activation. MSU crystals are phagocytosed by macrophages and induce lysosomal rupture, cathepsin release, potassium efflux, mitochondrial stress, mtROS generation, and mitochondrial DNA release. These stress signals converge on NLRP3 inflammasome assembly and caspase-1 activation, resulting in pro-IL-1β cleavage and mature IL-1β release. Glycolytic reprogramming and TCA cycle remodeling may support inflammatory activation through succinate accumulation, HIF-1α stabilization, and IL-1β transcription, while membrane lipid remodeling may further influence inflammasome-related signaling.

In gout-related models, MSU crystals can also induce lysosomal rupture, cathepsin release, mitochondrial ROS generation, and NLRP3/IL-1β activation, suggesting that TCA cycle remodeling and mitochondrial stress may jointly influence crystal-induced inflammatory responses (16, 33). In addition, citrate can be converted into acetyl-CoA by ATP-citrate lyase, providing substrates for lipid synthesis and histone acetylation, thereby linking metabolic remodeling to transcriptional regulation of inflammatory genes (34, 35). Conversely, itaconate and its derivatives can limit inflammatory responses by inhibiting SDH or activating NRF2 (36, 37), indicating that TCA cycle remodeling is not unidirectionally pro-inflammatory. Rather, its effects depend on the specific metabolite involved, the cellular state, and the stage of inflammation. **Figure 2** illustrates how glycolytic remodeling, TCA cycle alterations, and mitochondrial stress converge with MSU crystal-induced lysosomal injury to support macrophage NLRP3 inflammasome activation and IL-1β release.

### 3.3 Context-dependent roles of oxidative phosphorylation in gouty inflammation

Macrophage oxidative phosphorylation (OXPHOS) may exert distinct functions depending on cellular state. In homeostatic or tissue-resident macrophages, OXPHOS generally supports cell survival, tissue adaptation, phagocytic clearance, and maintenance of tissue homeostasis (38, 39). Synovial tissue-resident macrophages are also thought to form a protective barrier, thereby contributing to joint homeostasis and limiting unnecessary inflammatory responses (40, 41).

In the context of inflammatory activation, however, mitochondrial oxidative metabolism may have different implications. Mitochondrial respiration, increased electron transport chain pressure, ROS generation, and mitochondrial DNA release can be associated with NLRP3 inflammasome activation and pro-inflammatory cytokine production (32, 42–44). In MSU crystal stimulation models, ROS generation, lysosomal rupture, and NLRP3/IL-1β activation have also been observed, suggesting that mitochondrial stress may participate in crystal-induced inflammatory responses (16, 33).

Therefore, the role of OXPHOS in gout inflammation should be understood as state- and stage-dependent. It may support efferocytosis and tissue repair during homeostasis or the resolution phase, while during acute inflammation it may be associated with mitochondrial stress, ROS production, and inflammasome activation. Thus, OXPHOS should not be simply categorized as either pro-inflammatory or anti-inflammatory (39, 45).

Overall, macrophage metabolic reprogramming provides an important complementary perspective for understanding gouty inflammation. Enhanced glycolysis may support pro-IL-1β expression and inflammatory mediator synthesis, TCA cycle remodeling and mitochondrial stress may contribute to inflammasome activation, and OXPHOS may have distinct roles in homeostatic maintenance, inflammatory stress, and tissue repair. Future studies should further define the temporal sequence, cellular sources, and causal contributions of these metabolic changes.

## 4 Neutrophil metabolism, ROS, and NETs

Neutrophil infiltration is a hallmark of acute gout flares, mediating tissue-level amplification of inflammation rather than crystal sensing and inflammasome activation, which are primarily carried out by macrophages (17). IL-1β induces synovial fibroblasts and endothelial cells to produce chemokines and adhesion molecules, rapidly recruiting circulating neutrophils to the joint. At the inflammatory site, neutrophils contribute to acute inflammation by phagocytosing MSU crystals, releasing granular proteins, producing reactive oxygen species, and forming neutrophil extracellular traps (46, 47).

### 4.1 Glycolysis supports neutrophil effector functions

Neutrophil effector responses, including chemotaxis, adhesion, transendothelial migration, phagocytosis, and degranulation, require rapid energy support. Compared with macrophages, mature neutrophils contain relatively few mitochondria and have limited oxidative phosphorylation capacity; thus, their ATP production relies predominantly on glycolysis (48, 49). This metabolic feature enables neutrophils to exert effector functions rapidly in tissue environments characterized by hypoxia and abundant inflammatory mediators. Within the inflammatory microenvironment, hypoxic signaling may also prolong neutrophil survival through HIF-1α–related pathways, thereby sustaining their inflammatory activity (50).

Glucose metabolism not only provides ATP for neutrophils but also contributes to ROS generation. Upon activation, part of the glucose flux can enter the pentose phosphate pathway to generate NADPH, which supplies reducing equivalents for NADPH oxidase–mediated ROS production (51). In acute gout, MSU crystals can directly stimulate neutrophils, leading to crystal phagocytosis, granule protein release, ROS generation, and NET formation; together, these responses contribute to the amplification of local inflammation (46, 52). Therefore, in the context of gout, neutrophil glycolysis is better understood as a metabolic basis supporting migration, phagocytosis, degranulation, ROS generation, and NET formation, rather than as an independent initiating factor of gout flares.

### 4.2 NET formation and stage-dependent roles

NETs represent an important mechanism by which neutrophils participate in gouty inflammation. Structurally, NETs consist of a decondensed DNA scaffold decorated with histones, granular proteins such as myeloperoxidase and neutrophil elastase, and various antimicrobial molecules (53). MSU crystals can directly induce NET formation in neutrophils, thereby entrapping crystals and local inflammatory components within web-like structures. This process is usually accompanied by chromatin decondensation, translocation of granular proteins, ROS generation, and alterations in membrane integrity, and is therefore closely related to neutrophil activation status, the redox environment, and metabolic energy supply (52, 54).

During the early phase of acute inflammation, NETs are more likely to contribute to inflammatory amplification. Histones, proteases, and DNA components within NETs may act as local stimulatory factors, promoting activation of surrounding cells, inflammatory mediator release, and tissue injury (55). Upon MSU crystal stimulation, NET formation in neutrophils may further cooperate with ROS generation and granular protein release to amplify local inflammatory responses (46). From a metabolic perspective, glycolysis provides energy support for rapid neutrophil activation, while NADPH generated through the pentose phosphate pathway supplies reducing equivalents for NADPH oxidase-mediated ROS production (51). Thus, glucose metabolism and redox status may influence NET formation and its inflammatory consequences. In addition, although mitochondria are not the major source of ATP production in mature neutrophils, mitochondrial ROS and mitochondrial DNA may still serve signaling roles in certain forms of NET formation (56).

As inflammation progresses, the role of NETs may change. Aggregated NETs (aggNETs), formed after extensive neutrophil accumulation, can entrap MSU crystals and degrade cytokines and chemokines through proteases, thereby limiting the diffusion of inflammatory mediators and further neutrophil recruitment (57). This mechanism may partly contribute to the self-limited nature and resolution of acute gouty inflammation (58). However, this phenomenon should not be simply interpreted as NETs being inherently protective. Early or excessive NET formation may promote inflammation and tissue damage, whereas aggNETs formed under specific conditions and cleared appropriately may help with crystal sequestration and inflammatory containment (59, 60).

**Figure 3:**
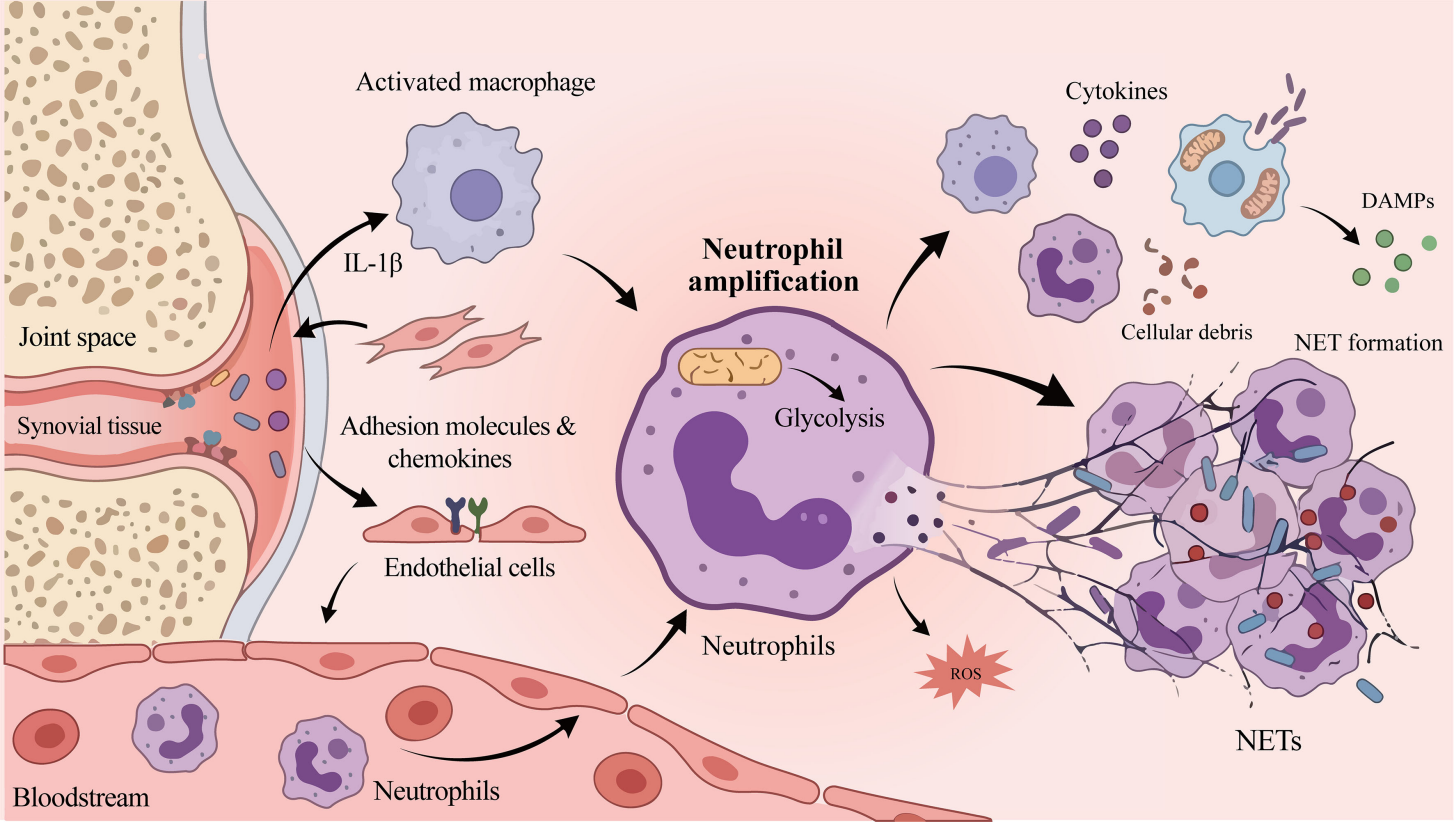
Neutrophil metabolism, ROS production, and NET formation in gouty inflammation. IL-1β released during MSU crystal-induced macrophage activation promotes chemokine and adhesion molecule expression by synovial and endothelial cells, thereby facilitating neutrophil recruitment from the bloodstream into the inflamed joint. Recruited neutrophils rely predominantly on glycolysis to support rapid effector functions, including migration, ROS production, and NET formation. Neutrophil-derived cytokines, cellular debris, damage-associated molecular patterns, ROS, and NETs contribute to local inflammatory amplification and tissue injury.

Therefore, the role of NETs in gout is clearly stage-dependent. If NET formation is excessive or clearance is insufficient, residual NET components may persistently stimulate synovial tissue, prolong inflammation, or promote tissue injury. Conversely, timely clearance of NETs, apoptotic neutrophils, and related cellular debris by macrophages may facilitate the transition from inflammatory amplification to resolution. Overall, neutrophil glycolysis, ROS generation, and NET formation jointly support the amplification of acute gouty inflammation, whereas subsequent aggNET formation and clearance efficiency may determine whether these effector structures continue to sustain inflammation or contribute to inflammatory containment and resolution. These neutrophil-centered processes are summarized in **Figure 3**. Future studies should integrate synovial fluid cells from patients with gout and dynamic disease-course samples to further clarify the relationships among neutrophil metabolic status, modes of NET formation, and inflammation resolution.

## 5 Metabolic regulation of inflammation resolution and recurrence susceptibility

Acute gout flares typically present with rapid onset and intense inflammation but are generally self-limiting (10). As the disease progresses, neutrophil infiltration decreases, inflammatory mediator levels decline, and joint redness, swelling, heat, and pain gradually resolve. This resolution is not merely a passive exhaustion of inflammatory stimuli; it involves active processes including apoptotic cell clearance, generation of pro-resolving mediators, local tissue repair, and re-establishment of immune homeostasis (60–62). Immunometabolism at this stage supports the transition of immune cells from effector functions toward clearance, repair, and restoration of homeostasis, rather than simply reflecting a decrease in pro-inflammatory metabolic activity.

Gout is also characterized by recurrent flares. Persistent hyperuricemia and unresolved MSU crystal burden form the basis of recurrence; however, flare timing and frequency are also influenced by short-term triggers such as alcohol consumption, high-purine diet, infection, trauma, surgery, fluctuations in uric acid during early urate-lowering therapy, and metabolic comorbidities (63–65). Therefore, gout recurrence cannot be explained solely by crystal burden; the basal activation state of host innate immune cells during intercritical periods must also be considered. Studies of trained immunity suggest that metabolic and epigenetic reprogramming can enhance monocyte/macrophage responses to subsequent stimuli (66, 67), and urate- or MSU-related stimulation may induce similar immune priming (68), although its clinical relevance to recurrent gout flares remains to be further clarified.

### 5.1 Macrophage clearance and lipid mediator switching during resolution

Macrophage clearance of apoptotic neutrophils is a key mechanism in the resolution of acute gout inflammation. After neutrophils complete their effector functions, they undergo apoptosis and are subsequently removed by macrophages through efferocytosis. Unlike pathogen phagocytosis, efferocytosis typically does not induce robust inflammation; instead, it promotes anti-inflammatory and pro-repair signaling. Failure to timely clear apoptotic neutrophils may result in secondary necrosis, releasing proteases, nucleic acids, and other damage-associated molecules, which can further stimulate synovial tissue and prolong inflammation (60, 62).

Efferocytosis imposes substantial metabolic demands on macrophages. Engulfing large numbers of apoptotic cells requires processing of exogenous lipids, cholesterol, and membrane components, thereby engaging lipid metabolism, cholesterol efflux, and mitochondrial oxidative metabolism to maintain clearance capacity (69–71). Unlike the glycolysis-enhanced, IL-1β–producing state observed during early inflammation, macrophages in the resolution phase generally require stable energy supply and effective mitochondrial quality control to support continuous clearance, lipid handling, and production of pro-repair mediators (72, 73). In this context, mitochondrial metabolism is more closely associated with clearance and tissue restoration rather than inflammasome activation.

Lipid mediator class switching is another critical mechanism driving inflammation resolution. During early acute inflammation, arachidonic acid metabolism produces prostaglandins and leukotrienes that promote vascular responses, pain sensitization, and neutrophil recruitment. As inflammation progresses, the local lipid mediator profile shifts toward pro-resolving species, including lipoxins, resolvins, protectins, and maresins (61, 62, 74). These mediators do not simply suppress inflammation; rather, they reduce further neutrophil recruitment, facilitate apoptotic cell clearance, and support tissue repair, enabling orderly termination of the inflammatory response (75).

In gout, pro-resolving lipid mediators and efferocytosis likely cooperate to drive the self-limiting nature of acute inflammation. The efficiency of NET and apoptotic neutrophil clearance, macrophage lipid handling capacity, and the local redox environment may all influence whether inflammation resolves successfully (57, 76). However, direct clinical evidence characterizing pro-resolving mediator dynamics, macrophage efferocytosis function, and metabolic features in the synovial tissue of gout patients remains limited; current data mainly suggest these processes contribute to resolution but are not yet sufficient to establish them as the sole or dominant mechanism underlying self-limiting flares.

### 5.2 Systemic metabolic abnormalities and recurrence susceptibility

Patients with gout commonly present with metabolic and cardiorenal comorbidities, including obesity, insulin resistance, dyslipidemia, hypertension, chronic kidney disease, and cardiovascular disease. These comorbidities not only affect urate production and excretion, but may also alter the basal responsiveness of innate immune cells through low-grade inflammation, oxidative stress, and metabolic stress (10, 68, 77). In the context of obesity and metabolic syndrome, adipose tissue inflammation, elevated free fatty acids, glycemic fluctuations, and gut-derived inflammatory signals may continuously act on monocytes and macrophages, making them more prone to an inflammatory primed state. This process is closely associated with TLR–NF-κB, NLRP3–IL-1β, PI3K–Akt–mTOR, and HIF-1α signaling pathways (78–80). Therefore, under conditions of systemic metabolic dysregulation, the same MSU crystal stimulus may induce stronger IL-1β production and neutrophil recruitment; however, this inference still requires further validation using cells from patients with gout and local joint-derived samples.

This state may be described as metabolic priming, with some features resembling trained immunity. Studies on trained immunity have shown that monocytes/macrophages and their progenitor cells can undergo metabolic and epigenetic reprogramming after an initial stimulus, enabling a stronger or more rapid inflammatory response upon subsequent stimulation (66). The metabolic basis of this process includes Akt–mTOR–HIF-1α-mediated enhancement of glycolysis, changes in TCA cycle intermediates, and alterations in histone modifications associated with inflammatory genes (81, 82). In the context of hyperuricemia and gout, soluble urate and MSU crystal-related stimuli have also been suggested to induce immune programming in myeloid cells, manifested as enhanced inflammatory responsiveness of monocytes/macrophages and altered epigenetic states (83, 84). Such a primed state is usually insufficient to independently trigger acute arthritis, but it may increase the responsiveness of innate immune cells to subsequent stimuli.

Persistent hyperuricemia, metabolic abnormalities, and repeated inflammatory stimulation may not only provide the conditions for MSU crystal formation, but also alter the responsiveness of innate immune cells during intercritical periods. When short-term triggers such as alcohol intake, purine-rich diet, infection, tissue injury, surgery, or urate fluctuations during the early phase of urate-lowering therapy occur, a relatively stable crystal deposition environment may be more readily converted into a clinical flare (64, 65).

Nevertheless, the role of trained immunity in gout should be interpreted cautiously. Much of the current evidence comes from models involving β-glucan, infection, oxidized low-density lipoprotein, or atherosclerosis, and cannot be directly equated with gout. Moreover, the epigenetic state of peripheral blood monocytes may not fully represent that of joint-resident macrophages or the synovial microenvironment. Systemic metabolic abnormalities, hyperuricemia, and repeated inflammatory stimulation may alter innate immune responsiveness to MSU crystals, but whether persistent metabolic or epigenetic reprogramming contributes to flare recurrence requires validation in longitudinal patient samples and joint-derived cells.

## 6 Therapeutic implications

Current gout management remains centered on long-term serum urate control and suppression of acute inflammation during flares. Urate-lowering therapy reduces MSU crystal formation and promotes gradual dissolution of pre-existing deposits, thereby decreasing the risk of recurrent flares and tophus progression. Acute anti-inflammatory treatment mainly includes nonsteroidal anti-inflammatory drugs, colchicine, glucocorticoids, and, in selected patients with refractory disease or contraindications to standard therapies, IL-1 pathway inhibitors (85). Immunometabolism research does not alter this basic treatment framework. Rather, its major value lies in helping to explain the heterogeneity of inflammatory responses in gout, including why some patients experience more frequent flares, why the intensity and duration of acute inflammation vary, and why the capacity for inflammation resolution differs among individuals.

Mechanistic studies suggest that glycolysis, TCA cycle remodeling, mitochondrial stress, ROS generation, and lipid mediator switching may influence the magnitude and duration of MSU crystal-induced inflammation. These findings provide new insights into gout inflammation, but most remain at the stage of basic research or preclinical exploration. Directly targeting glycolysis, mitochondrial metabolism, or lipid metabolism poses substantial challenges because these metabolic processes are broadly involved in normal immune defense, inflammation resolution, tissue repair, and systemic metabolic homeostasis. The same metabolic pathway may have distinct roles across different cell types and disease stages. For example, glycolysis may support cytokine production and neutrophil effector responses during early inflammation, whereas mitochondrial metabolism and lipid handling may contribute to efferocytosis, pro-resolving responses, and tissue repair during later stages. Therefore, any attempt to target metabolic pathways therapeutically must carefully consider cell type, disease stage, timing of intervention, and safety.

In addition to suppressing inflammatory initiation, the mechanisms governing inflammation resolution warrant further investigation. Acute gout is characteristically self-limiting, suggesting the presence of endogenous mechanisms that terminate inflammation. Apoptotic neutrophil clearance, specialized pro-resolving lipid mediators, mitochondrial quality control, and restoration of local tissue homeostasis may all contribute to the timely resolution of inflammation (60). In experimental gout models, endogenous pro-resolving pathways such as Annexin A1 have been shown to promote timely resolution of MSU crystal-induced inflammation (76), while aggregated NETs may also contribute to inflammatory containment by degrading cytokines and chemokines (57). However, clinical evidence for pro-resolving mediators or metabolic modulation strategies in gout remains insufficient. Their efficacy, optimal timing of administration, target populations, and long-term safety all require further validation.

Systemic metabolic management remains an important component of comprehensive gout care. Weight control, alcohol restriction, dietary modification, glycemic and lipid control, renal protection, and cardiovascular risk reduction may all help reduce gout flares and improve long-term outcomes (77). However, the benefits of these interventions cannot be simply attributed to a single immunometabolic mechanism, as they simultaneously affect urate production, urate excretion, crystal burden, systemic low-grade inflammation, and multiple comorbid conditions. Future studies evaluating the clinical value of immunometabolism in gout management should integrate it with serum urate control, changes in crystal burden, and comorbidity management.

Several key questions remain unresolved in gout immunometabolism research. First, the temporal relationship between metabolic changes and inflammatory flares needs to be clarified, because enhanced glycolysis, mitochondrial ROS production, or TCA cycle remodeling may occur either before inflammatory activation or as downstream consequences of it. Second, cell and tissue specificity should be emphasized. Peripheral blood monocytes, synovial fluid macrophages, synovial tissue-resident macrophages, and neutrophils have distinct metabolic states and functional properties, and should not be treated as interchangeable cell populations (41). Third, different stages of disease should be clearly distinguished. The same metabolic pathway may have different implications during acute flares, inflammation resolution, and intercritical periods. Future studies using synovial fluid, synovial tissue, and longitudinal patient samples are needed to determine whether these pathways have practical value for risk stratification, recurrence prevention, or adjunctive therapy.

## 7 Conclusion

In summary, immunometabolism provides a regulatory perspective rather than an alternative pathogenic model for gout. Metabolic adaptations in macrophages and neutrophils may influence the magnitude of MSU crystal-induced inflammation, the efficiency of resolution, and susceptibility to recurrent flares. Future studies should move beyond stimulus-based *in vitro* observations and define the temporal sequence, cellular specificity, and causal relevance of these pathways in patient-derived synovial fluid, synovial tissue, and longitudinal samples.
